# Current and future therapies for follicular lymphoma

**DOI:** 10.1186/s40164-024-00551-1

**Published:** 2024-08-22

**Authors:** Pier Luigi Zinzani, Javier Muñoz, Judith Trotman

**Affiliations:** 1 IRCCS Azienda Ospedaliero-Universitaria di Bologna Istituto di Ematologia Seràgnoli, Dipartimento di Scienze Mediche e Chirurgiche, Università di Bologna, Bologna, Italy; 2 Division of Hematology and Oncology, Mayo Clinic, Phoenix, AZ USA; 3 Concord Repatriation General Hospital, University of Sydney, Concord, NSW Australia

**Keywords:** Follicular lymphoma, Indolent B-cell lymphoma, CAR-T cell therapy, BTK inhibitor

## Abstract

Follicular lymphoma (FL) is an indolent, germinal center B cell–derived lymphoid neoplasm, for which recent advances in treatment have substantially improved patient survival. However, FL remains an incurable and heterogeneous disease, with groups of patients experiencing early disease progression, histologic transformation, or a high risk of treatment-related toxicity. Additionally, FL is a continually relapsing disease, and response rates and disease-control intervals decrease with each subsequent line of therapy. In this review, we explore the current treatment landscape for relapsed or refractory FL and promising therapies in development, highlighting the efficacy and potential risks of each treatment. We provide a real-world perspective on the unmet needs of patients with FL. Novel therapeutic approaches in development offer a wide array of options for clinicians when treating relapsed or refractory FL. A nuanced approach is required to address the needs of individual patients, taking into consideration both the risks and benefits of each treatment option, as well as patient preferences.

## Background

Follicular lymphoma (FL) is the second most common form of non-Hodgkin lymphoma (NHL), accounting for approximately 5% of all hematologic neoplasms [1]. In Western countries, the age-standardized incidence rate is 2 to 4 FL cases per 100,000 people per year, with an estimated 13,960 new cases diagnosed in the US in 2016 and approximately 2,220 and 2,500 new cases diagnosed per year in the UK and France, respectively [1]. Developments in the treatment of FL in the new millennium, primarily the introduction of anti-CD20 therapies, have led to substantial improvements in survival [2, 3].

Despite advances in treatment, FL remains an incurable disease with continuous patterns of relapse and progressively shorter disease-control intervals with each line of treatment. Although most patients have indolent disease and remain asymptomatic for decades, with many dying with lymphoma rather than because of it, some patients have an aggressive clinical course, and lymphoma remains the most common cause of death [3]. The clinical heterogeneity of FL poses a challenge to clinicians, who need to consider age, comorbidities, likelihood of relapse, and treatment accessibility when deciding on the most appropriate treatment strategy at each time point. Here, we provide an overview of the current treatment landscape for patients with relapsed/refractory (R/R) FL and a perspective on real-world clinical considerations in addressing the unmet needs of specific patient groups.

## Characteristics and mechanisms of disease

FL is an indolent, germinal center B cell–derived lymphoid neoplasm. The hallmark t(14;18)(q32;q21) translocation, in which the *BCL2* gene is placed under the transcriptional control of the *IHC* gene enhancer, is present in 65–85% of cases, leading to overexpression of the antiapoptotic BCL2 protein [4, 5]. Components of the B-cell receptor signaling pathway, including phosphoinositide 3-kinase (PI3K), Bruton tyrosine kinase (BTK), and spleen tyrosine kinase, are frequently activated in FL, warranting the development and use of kinase inhibitors for treating FL [6]. Additionally, genes with a role in posttranslational modification of histones are frequently mutated in FL, including histone methyltransferases (HMTs), *KMT2D*,* KMT2C*, and *EZH2*, as well as histone acetyltransferases *CREBBP* and *EP300* [7]. Together, these characteristic mutations highlight key therapeutic targets in the development of treatment for FL.

### Clinical presentation

FL is a low-grade lymphoma for which systemic treatment may be deferred until symptoms develop [8]. FL is a biologically heterogeneous disease with a diverse range of clinical presentations, each posing a unique challenge to clinicians. The majority of patients with FL present with an indolent disease course and higher stage disease [9]. Patients with FL also have a generally favorable prognosis in the rituximab era, with 10-year overall survival (OS) rates of 79.8% and 76.6% in French and US cohorts, respectively, and 69.9% of patients remaining event free for 2 years, based on a pooled analysis of patients with newly-diagnosed FL [3].

Despite the generally indolent nature of FL, subsets of this patient population have more aggressive disease. Histologic transformation of FL to a high-grade, aggressive lymphoma—commonly diffuse large B-cell lymphoma—is associated with a poor prognosis and lower survival rates [10]. The 10-year cumulative hazard of histologic transformation has significantly decreased from 8.7% (95% CI, 7.2–10.6) in patients who did not receive rituximab to 5.2% (95% CI, 4.5–6.2) in those who did and is as low as 3.6% (95% CI, 2.3–5.5) in those who received both rituximab induction and maintenance therapy. However, survival after transformation does not differ between patients who received rituximab and those who did not [11]. Analysis of US and French FL cohorts demonstrated that death was most commonly caused by lymphoma, with a 10-year cumulative incidence of 10.3% (95% CI, 8.6–12.2), and the majority of patients who died due to lymphoma had transformed disease [3].

Approximately 20% of patients experience disease progression within 24 months of first-line treatment (POD24) [12], with significantly lower survival rates [12]. Patients with POD24 had 2-year and 5-year OS rates of 68% and 50%, respectively, versus 97% and 90% in patients without POD24 [13]. Pooled analysis of 13 randomized clinical trials showed that male sex, Eastern Cooperative Oncology Group performance status of ≥ 2, intermediate- or high-risk FLIPI score, and an elevated beta 2 microglobulin level each correlated with a higher risk of POD24 [14]. Patients with early progression, particularly those who experience histologic transformation, represent a high-risk population with needs that must be addressed through biomarker and treatment development [15].

### Current treatment landscape for R/R FL

Frontline therapy for FL is well defined, with most patients achieving a sustained response to chemoimmunotherapy for many years [16, 17]. Patients with localized FL can also be treated either with radiotherapy alone or in combination with immunochemotherapy [18, 19]. RT alone may be an appropriate treatment for localized relapsed FL. However, in general the prognosis in patients with R/R FL, particularly those who have received 2 prior therapies, remains poor, with a median PFS of 17 months (95% CI, 15–19) and a 5-year OS of 75% (95% CI, 70–79) reported in the LEO CReWE study [20]. Patients who experience POD24 or histologic transformation are at high risk for aggressive disease and early mortality. Patients with multiple relapses require novel treatment to address poor response to previous lines of treatment. The National Comprehensive Cancer Network guidelines for B-cell lymphomas and the European Society for Medical Oncology Clinical Practice Guidelines outline the broad range of options available for treatment of R/R FL [21, 22]. When deciding between therapeutic options for advanced R/R FL, clinicians need to weigh the risks and benefits of available options for each specific patient, considering age, comorbidities, previous lines of therapy, and disease burden, as well as treatment availability by region and patient preference.

#### Second-line treatment strategies

When relapse or progression is suspected, a confirmatory biopsy is recommended to identify transformation to aggressive lymphoma. In asymptomatic patients with confirmed follicular histology, the watch-and-wait approach is acceptable. In the second-line setting, treatment regimens usually used as frontline therapy may be recommended if they have not already been used. Rituximab maintenance may also be beneficial after standard frontline treatments such as bendamustine plus rituximab (BR), particularly in those who achieve a partial response with BR [23]. Although not used as commonly as immunochemotherapy, autologous stem cell transplant (SCT) is also a viable treatment option with potential benefit in those with early treatment failure with immunochemotherapy [24]. Radioimmunotherapy is another treatment option for patients with FL. The radio-immunoconjugate, yttrium-90 ibritumomab tiuxetan, is approved in R/R low-grade FL and has demonstrated high response rates in patients with untreated (objective response rate [ORR], 100%; complete response [CR] rate, 93%) or R/R (ORR, 93%; CR rate, 73%) FL [25]. The National Comprehensive Cancer Network recommends BR or bendamustine plus obinutuzumab if bendamustine has not been used as frontline therapy [21]. Cyclophosphamide, doxorubicin, vincristine, and prednisone (CHOP) or cyclophosphamide, vincristine, and prednisone combined with obinutuzumab or rituximab is also recommended. Findings from a retrospective analysis of claims data in the US between 2008 and 2016 showed that the most commonly prescribed second-line regimens were rituximab monotherapy (34%); BR (27%); and rituximab plus cyclophosphamide, vincristine, and prednisone (9%) [26]. Rituximab plus lenalidomide (R2), which showed a significant improvement in PFS compared with rituximab alone in patients with R/R indolent lymphoma in the phase 3 AUGMENT trial, is also a preferred second-line treatment [27]. Both standard chemoimmunotherapy and R2 have unique toxicity profiles that should be considered when deciding on appropriate treatment options for patients, particularly those who are elderly and/or have comorbidities.

#### Third-line and beyond treatment strategies

In the third and subsequent lines of therapy, the second-line treatment options listed in the previous section may be considered if they have not previously been used. Data from a multicenter, observational study, collected from medical records of patients in the US with non-transformed grade 1-3a FL diagnosed between 2002 and 2018 who received third-line or later systemic therapy, showed variable treatment regimens and sequencing [20]. The most common index therapy was chemoimmunotherapy (30%). Other treatment types included dose-dense salvage chemotherapy and/or cellular therapy (21%), anti-CD20 monotherapy (12%), experimental therapy with or without anti-CD20 therapy (9%), lenalidomide with or without anti-CD20 therapy (8%), PI3K inhibitors (now withdrawn from the US market) with or without anti-CD20 therapy (6%), autologous SCT (10%), allogeneic SCT (3%), and other treatments (13%) [20]. Similarly, CR rates varied, ranging from 10% with PI3K inhibitors to 61% with salvage chemotherapy and/or cellular therapy. Despite high response rates, median duration of response (DOR) and median PFS were < 2 years, suggesting an increasing need for more effective treatment strategies [20].

Similarly, the retrospective, real-world SCHOLAR-5 study examined treatment patterns and outcomes in patients in the US and Europe receiving third-line or later treatment [28]. Treatment patterns differed by region, with anti-CD20 monotherapy (20% vs. 2%) and R2 and other immunomodulatory drugs (12% vs. 6%) more commonly prescribed in the US vs. Europe and SCT more commonly prescribed in Europe vs. the US (18% vs. 0% for autologous SCT; 5% vs. 0% for allogenic SCT). More patients received experimental treatments in the fourth line than in the third line, regardless of region. This study showed diminishing response rates, DOR, and OS with each subsequent line of therapy, as well as a lack of a well-defined clinical approach for patients with multiple FL relapses, highlighting the heterogeneity and unmet needs of patients with advanced disease [28].

With the growing body of evidence for new treatment options, the treatment landscape for R/R FL is continually evolving. Autologous and allogeneic SCT can be used for treating relapsed FL, but their use has decreased over time as better-tolerated therapeutic options have emerged. With targeted agents and novel compounds with lower toxicity in development, the use of SCT may be considered in younger and/or fit patients, depending on approved treatment options by region. Additionally, treatment options that may have been promising in theory have demonstrated limited clinical activity. Given its role in FL development, BCL2 was considered a promising therapeutic target; however, the combination of the BCL2 inhibitor venetoclax and BR demonstrated no significant efficacy improvement in patients with R/R FL compared with BR alone, while increasing toxicity [29]. Similarly, despite the inherent immunosensitivity of FL, the immune checkpoint inhibitor nivolumab was not effective in patients with R/R FL [30]. The anti–PD-1 antibody pembrolizumab combined with rituximab showed efficacy in patients with R/R FL, with an ORR of 67% and CR rate of 50% [31]. Although promising, this study only included rituximab-sensitive patients, and this treatment may not demonstrate the same efficacy in those with rituximab-refractory disease [31]. Still, several promising treatment options are in development for R/R FL, as demonstrated by recent and ongoing studies, and novel therapeutic options have been recently approved.

Later lines of therapy recommended by current guidelines and regulatory approvals may include novel therapeutic options, such as the EZH2 inhibitor tazemetostat (approved in the US), BTK inhibitor combinations (zanubrutinib + obinutuzumab, approved in the EU and US), chimeric antigen receptor (CAR) T-cell therapies axicabtagene ciloleucel and tisagenlecleucel, and the bispecific antibody (BsAb) mosunetuzumab-axgb [21]. These therapies are described in more detail below.

### Recently approved therapies for R/R FL

#### BsAbs

T-cell–engaging BsAbs are designed to simultaneously bind antigens on the surface of tumor cells and CD3 on T cells, thereby directing T cells to engage and eliminate tumor cells [32]. Mosunetuzumab, a CD3- and CD20-targeted BsAb, is approved for patients with R/R FL who have received ≥ 2 prior lines of treatment, based on results from the phase 2 GO29781 trial [33]. Patients in this study who achieved CR received mosunetuzumab for eight 21-day cycles with step-up dosing in cycle 1; patients with a partial response or stable disease continued treatment for up to 17 cycles. In the 90 patients receiving mosunetuzumab, the independent review committee–assessed CR rate was 60% (95% CI, 49.1–70.2), which was significantly higher than the historical control of 14%, thereby meeting the primary endpoint. The median DOR in patients who responded was 22.81 months (95% CI, 9.7-not reached). By independent review committee assessment, the median PFS was 17.9 months (95% CI, 9.7–not reached) [33]. Cytokine release syndrome (CRS) occurred in 44% of patients, predominantly grade 1/2, and no fatal adverse events (AEs) were reported [34]. Subgroup analysis using updated results from this study with ≥ 3 year–follow-up showed consistent ORR and CR rates between patients with POD24 (ORR, 80.9%; CR rate, 59.6%), fourth line of therapy or later (ORR, 72.7%; CR rate, 54.5%), and in those aged ≥ 65 years (ORR, 83.3%; CR rate, 66.7%) compared with the overall population (ORR, 77.8%; CR rate, 60.0%) [35]. Given its efficacy and safety profile, mosunetuzumab was the first BsAb to be approved for use in R/R FL in the US and EU. The safety and efficacy of mosunetuzumab combined with lenalidomide are being compared with those of R2 in patients with R/R FL with ≥ 1 prior line of therapy in the ongoing randomized, open-label, phase 3 CELESTIMO trial [36].

#### BTK inhibitors

BTKs are downstream components of the B-cell–receptor signaling pathway, which regulates B-cell development, and are a common target in B-cell malignancies. BTK inhibitors have demonstrated clinically impactful efficacy in chronic lymphocytic leukemia, mantle cell lymphoma, marginal zone lymphoma, and other indications [37]; however, historically they have not shown similar promise as a monotherapy in R/R FL. The first-generation BTK inhibitor ibrutinib showed modest results as a monotherapy in the phase 2 DAWN study in patients with R/R FL (ORR, 21%; CR rate, 11%) [38]. In the R/R setting, the addition of ibrutinib to chemoimmunotherapy (BR or rituximab-CHOP) did not significantly improve PFS compared with chemotherapy alone, while toxicity increased [39].

Zanubrutinib, a next-generation BTK inhibitor, was evaluated in a phase 1/2, open-label, single-arm trial in patients with R/R indolent NHL [40]. With zanubrutinib monotherapy, the R/R FL cohort demonstrated an ORR of 36.4% (95% CI, 20.4–54.9) and a CR rate of 18.2% (95% CI, 7-35.5). Zanubrutinib was studied in combination with obinutuzumab in the phase 2 ROSEWOOD study comparing the efficacy and safety of zanubrutinib plus obinutuzumab (ZO) with those of obinutuzumab monotherapy in patients with R/R FL. The median number of prior treatments was 3 (range, 2–11). ZO demonstrated improved ORR and CR rates versus obinutuzumab alone (ORR, 69% [95% CI, 61–76] vs. 46% [95% CI, 34–58]; CR rates, 39% and 19%, respectively) [41]. The median time to first response was 2.8 months both with ZO (range, 2.0–23.0) and obinutuzumab (range, 2.5–6.5). However, median DOR was not reached (95% CI, 25.3–not evaluable [NE]) with ZO and 14 months (95% CI, 9.2–25.1) with obinutuzumab. Median PFS was 28 months (95% CI, 16.1–NE) with ZO versus 10.4 months (95% CI, 6.5–13.8) with obinutuzumab alone. At least 1 any-grade AE was reported in 94% of patients who received ZO, with grade ≥ 3 AEs reported in 63% of all patients. The most common AEs were thrombocytopenia, neutropenia, diarrhea, fatigue, and constipation. Obinutuzumab-based treatment in FL has shown better PFS rates and similar ORRs compared with rituximab-based treatment; however, rates of AEs, especially infusion-related events, were higher with obinutuzumab-based treatment [16]. Notably, in ROSEWOOD, pyrexia (11% vs. 20%) and infusion-related reactions (3% vs. 10%) occurred less frequently in patients treated with ZO versus obinutuzumab alone [41]. In a pooled safety analysis, zanubrutinib was well tolerated with a safety profile consistent with other BTK inhibitors [42].

Based on these results, BTK inhibitors have limited utility as monotherapy but may be effective in combination with other therapies. ZO was recently approved in the EU and US for the treatment of patients with R/R FL who have received ≥ 2 prior lines of therapy, based on results of the ROSEWOOD study [41]. A comparison of ZO and R2 in patients with R/R FL is ongoing in the randomized phase 3 MAHOGANY trial (NCT05100862). Other BTK inhibitors have been investigated in R/R FL in combination with other therapies, including ibrutinib plus venetoclax [43], as well as acalabrutinib monotherapy, acalabrutinib plus rituximab, and acalabrutinib plus R2 [44].

#### CAR T-cell therapy

CAR-T cells are autologous T lymphocytes genetically modified to express CARs, which target specific tumor antigens, on their surface. Axicabtagene ciloleucel and tisagenlecleucel are both CD19-directed CAR T-cell therapies approved in the US and EU for patients with R/R FL. Axicabtagene ciloleucel is approved for use after ≥ 2 prior lines of therapy in the US and ≥ 3 prior lines of therapy in the EU, based on the results of the phase 2 ZUMA-5 study [45]. In a long-term follow-up analysis of ZUMA-5 with a median follow-up of 41.7 months (range, 32.7–57.4), the ORR and CR rate were 94% (95% CI, 88–97) and 79%, respectively, in patients with FL with a median of 3 (range, 1–10) prior lines of treatment; median DOR was 38.6 months (95% CI, 29.0-NE), and median PFS was 40.2 months (95% CI, 28.9-NE) [46]. No new safety signals were observed since the primary analysis, in which CRS occurred in 78% of patients with R/R FL, with most cases being grade 1/2 and 6% being grade ≥ 3. Neurological events occurred in 56% of patients with FL, with the majority being grade 1/2 and 15% being grade ≥ 3 [45, 46]. Of 127 patients with FL, 4 died due to treatment-related AEs. Axicabtagene ciloleucel has demonstrated high efficacy in R/R FL; however, these important safety considerations limit widespread use in this patient population.

Tisagenlecleucel is approved for use in adult patients with R/R FL after ≥ 2 prior lines of therapy based on results of the single-arm phase 2 ELARA trial [47]. In this ongoing study, the efficacy of tisagenlecleucel was evaluated in adult patients with R/R FL [47]. The ORR was 86% (95% CI, 77.5–92.4), and the CR rate was 69% (95% CI, 58.8–78.3). At least 1 grade ≥ 3 AE occurred in 78% of patients, most commonly neutropenia. CRS occurred in 49% of all patients, with no patients reporting grade ≥ 3 CRS [47]. Any-grade neurological events occurred in 37.1% and immune effector cell–associated neurotoxicity syndrome (ICANS) in 4.1% of all patients. The estimated 12-month PFS rate was 86% (95% CI, 74–92) in patients who achieved CR and 67% (95% CI, 56–76) in the overall population. Extended follow-up analysis with a median follow-up of 29 months showed consistent efficacy (ORR, 86.2%; CR rate, 68.1%), with no new safety signals or treatment-related deaths [48].

Lisocabtagene maraleucel is approved in the US for patients with R/R FL after ≥ 2 prior lines of therapy, following the results of the phase 2 TRANSCEND-FL study [49]. In this study, patients with POD24 who received lisocabtagene maraleucel as second-line therapy had an ORR of 96% (95% CI, 78.1–99.9), all of which were CRs. Those receiving lisocabtagene maraleucel as third-line or later therapy had an ORR of 97% (95% CI, 91.6–99.4), with a CR rate of 94% (95% CI, 87.5–97.8). At least 1 grade ≥ 3 AE occurred in 75% of patients, most commonly neutropenia (58%), anemia (10%), and thrombocytopenia (10%). CRS occurred in 58% of patients, with 1 patient (1%) experiencing grade 3 CRS [49]. Any-grade neurological events occurred in 15% of patients (2% grade 3; no grade > 3). The high efficacy demonstrated by axicabtagene ciloleucel, tisagenlecleucel, and lisocabtagene maraleucel in R/R FL provides additional options for patients deemed appropriate for CAR-T therapy.

#### EZH2 inhibitors

Genes encoding chromatin-modifying enzymes, such as HMTs, are frequently mutated in NHL [50]; somatic mutation of the *EZH2* HMT occurs in > 25% of patients with FL [51]. Tazemetostat is a first-in-class oral *EZH2* inhibitor approved in the US for the treatment of patients with R/R FL who have received ≥ 2 prior lines of systemic therapy and have an *EZH2* mutation, as well as patients with R/R FL who have no satisfactory alternative treatment option, regardless of *EZH2* status [52]. Tazemetostat was evaluated in an open-label, single-arm, phase 2 study in patients with R/R FL. The ORR was 69% (95% CI, 53–82) in patients with mutated *EZH2* and 35% (95% CI, 23–49) in patients with wild-type *EZH2*, with a 13% and 4% CR rate, respectively [53]. Median PFS was 13.8 months (95% CI, 10.7–22.0) in patients with mutated *EZH2* and 11.1 months (95% CI, 3.7–14.6) in those with wild-type *EZH2*. The most common grade ≥ 3 treatment-related AEs were thrombocytopenia (3%), neutropenia (3%), and anemia (2%) [53]. Tazemetostat presents another oral therapeutic option that uses a novel mechanism with a well-tolerated safety profile. The combination of tazemetostat with R2 for R/R FL is being investigated in the phase 3 part of the SYMPHONY-1 study [54, 55].

These recently approved treatments provide options for patients with R/R FL who may not respond to commonly used treatment options. Novel therapeutics evaluated in recent and ongoing trials, which may lead to approval of additional treatment options, are summarized in the following section.

### Summary of recent clinical trials in R/R FL

Recent clinical trials of other therapeutic options for patients with R/R FL, specifically those with high-risk disease or multiple relapses, are briefly described and are summarized in Table 1.

**Table 1:** Clinical outcomes with therapies in recent clinical trials in R/R FL

Therapy	Trial identifier	Trial phase	Publication	Follow-up, median, months	Number of patients with R/R FL	Number of prior therapies, median (range)	Efficacy outcomes reported (95% CI)
Mosunetuzumab	NCT02500407	2	Budde et al. 2022 [33]	18.3	90	3 (2–4)	CRR: 60% (49.1–70.2)
Ibrutinib	DAWN (NCT01779791)	2	Gopal et al. 2018 [38]	27.7	110	3 (2–13)	ORR: 20.9% (13.7–29.7) CRR: 11% (5.8–18.3 mPFS: 4.6 months (2.8–5.5)
Ibrutinib + BR/R-CHOP	SELENE (NCT01974440)	3	Nastoupil et al. 2023 [39]	84.0	174	-	mPFS: 38.4 months (24.2–49.4)
Ibrutinib + venetoclax	NCT02956382	2	Ujjani et al. 2020 [43]	-	14	1 (1–8)	ORR: 64% (35–87) CRR: 21% mPFS: 8.6 months (2.7-NE)
Acalabrutinib ± rituximab/R2	NCT02180711	1b	Strati et al. 2022 [44]		Acalabrutinib: 12 Acalabrutinib + rituximab: 13 Acalabrutinib + R2: 29	Acalabrutinib: 2 (1–5) Acalabrutinib + rituximab: 1 (1–5) Acalabrutinib + R2: not reported	Acalabrutinib: ORR: 33.3% (9.9–65.1) CRR: 8.3% Acalabrutinib + rituximab: ORR: 33.3% (9.9–65.1) CRR: 16.7% Acalabrutinib + R2: ORR: 80.8% (60.6–93.4) CRR: 30.8%
Zanubrutinib	NCT02343120	1/2	Phillips et al. 2022 [40]	32.8	33	3 (1–8)	ORR: 36.4% (20.4–54.9) CRR: 18.2% (7.0-35.5) mPFS: 10.4 months (7.7–22.9)
Zanubrutinib + obinutuzumab	ROSEWOOD (NCT03332017)	2	Zinzani et al. 2023 [41]	20.2	145	3 (2–11)	ORR: 69% (34–58) CRR: 39% mPFS: 28.0 months (16.1-NE)
Axicabtagene ciloleucel	ZUMA-5 (NCT03105336)	2	Neelapu et al. 2024 [46]	41.7	127	3 (1–10)	ORR: 94% (88–97%) CRR: 79% mPFS: 40.2 months (28.9-NE)
Tisagenlecleucel	ELARA (NCT03568461)	2	Fowler et al. 2022 [47]	16.85	97	4 (2–13)	ORR: 86.2% (77.5–92.4) CRR: 69.1 (58.8–78.3) PFS: NE (12.3-NE)
Tazemetostat	NCT01897571	2	Morschhauser et al. 2020 [53]	*EZH2* mutated: 22.0 *EZH2* WT: 35.9	*EZH2* mutated: 45 *EZH2* WT: 54	-	*EZH2* mutated: ORR: 69% (53–89) CRR: 13% mPFS: 13.8 months (10.7–22.0) *EZH2* WT: ORR: 35% (23–49) CRR: 4% mPFS: 11.1 months (3.7–14.6)
Tazemetostat + R2	SYMPHONY-1 (NCT04224493)	1b	Salles et al. 2023 [55]	22.5	44	1 (1–4)	ORR: 90.9% CRR: 54.8%
Odronextamab	ELM-2 (NCT03888105)	2	Villasboas et al. 2023 [56]	26.6	140	3 (2–13)	ORR: 80% CRR: 72% mPFS: 20.7 months (16.7–26.5)
Epcoritamab	EPCORE NHL-1	1/2	Linton et al. 2024 [58]	17.4	128	3 (2–9)	ORR: 82.0% (74.3–88.3) CRR: 62.5% (53.5–70.0)
AZD0486	NCT04594642	1	Devata et al. 2024 [60]	10.5	29	3 (2–9)	At doses of ≥ 0.8 mg: ORR: 92% CRR: 79%
Polatuzumab vedotin + rituximab	ROMULUS (NCT01691898)	2	Morschhauser et al. 2019 [61]	NE	22	2 (2–4)	ORR: 70% (46–88) CRR: 40% (23–68)
Polatuzumab vedotin + obinutuzumab	ROMULUS (NCT01691898)	1b/2	Phillips et al. 2022 [73]	12.3	36	-	ORR: 66.7% (90% CI 51.7–79.5) CRR: 36.1% (90% CI 22.9–51.2)
Polatuzumab vedotin + obinutuzumab + venetoclax	NCT02611323	1b	Lasater et al. 2023 [74]	-	33	3 (1–7)	ORR: 75.8% CRR: 57.6%
Polatuzumab vedotin + obinutuzumab + lenalidomide	NCT02600897	1b/2	Diefenbach et al. 2021 [75]	26.7	46	3 (IQR 2–4)	ORR: 76% (64–86) CRR: 63% (50–75) mPFS: NE
Loncastuximab tesirine	NCT04998669	2	Alderuccio et al. 2023 [62]	4.8	26	1 (1–6)	ORR: 95.2% CRR: 66.7%
Relmacabtagene autoleucel	RELIANCE (NCT04089215)	2	Song et al. 2022 [64]	11.7	28	-	6-month ORR: 92.6% (75.7–99.1) 6-month CRR: 77.8% (57.7–91.4) mPFS: NR
Lisocabtagene maraleucel	TRANSCEND FL (NCT04245839)	2	Morschhauser et al. 2024 [49]	18.1	130	2 (1–10)	ORR: 93% (87.2–96.5) CRR: 91% (84.5–94.9) mPFS: NR (21.4-NR)
Pirtobrutinib	BRUIN (NCT03740529)	1/2	Shah et al. 2023 [65]	18.4	48	3 (1–12)	ORR: 50.5% (35.2–64.8) CRR: 14.6%

BR, bendamustine plus rituximab; CRR, complete response rate; FL, follicular lymphoma; mPFS, median progression-free survival; NE, not estimable; ORR, objective response rate; R2, rituximab plus lenalidomide; R-CHOP, rituximab plus cyclophosphamide, doxorubicin, vincristine, and prednisone; R/R, relapsed/refractory; WT, wild type

#### BsAbs

Recent results from the phase 2 ELM-2 study evaluating the CD3- and CD20-targeted BsAb odronextamab demonstrated that patients with R/R FL who had received a median of 3 (range, 2–13) prior lines of treatment had an ORR of 80% and a CR rate of 72% with a median DOR of 21.7 months. All-grade CRS was reported in 55% of patients. In patients receiving the 0.7/0.4/20-mg step-up regimen, 98% of CRS events were grade 1/2, and only 1 low-grade ICANS event was reported [56].

The anti-CD20xCD3 BsAb epcoritamab was evaluated as a monotherapy in patients with R/R CD20 + B-cell NHL in a phase 1/2 study [57]. In the phase 1/2 EPCORE NHL-1 study, 128 patients in the R/R FL cohort (≥ 2 prior lines of therapy) treated with epcoritamab had an ORR of 82.0% (95% CI, 74.3–88.3) and a CR rate of 62.5% (95% CI, 53.5–70.9) [58]. The most common grade 3/4 treatment-emergent AE was neutropenia (25%), grade 1/2 CRS was reported in 65% of patients, and grade 3 CRS was reported in 2%. ICANS was reported in 6% of patients, none of which were grade ≥ 3 [58]. Epcoritamab is currently under evaluation in combination with R2 compared with R2 alone in patients with R/R FL in the ongoing phase 3 EPCORE FL-1 trial [59].

Updated data from an ongoing phase 1 dose-escalation study (NCT04594642) evaluating a CD3- and CD19-targeted BsAb, AZD0486, in patients with R/R FL with a median of 3 (range, 2–9) prior lines of therapy showed an ORR and CR rate of 92% and 79%, respectively, in patients who received doses of ≥ 0.8 mg, and 95% and 84%, respectively, in those who received doses of ≥ 2.4 mg [60]. The most common grade 3/4 treatment-related AEs were lymphopenia (34%), neutropenia (14%), ICANS (7%), and hypertension (7%). Infections occurred in 15 (52%) patients, of which 14% were grade ≥ 3. The findings of ongoing studies of BsAbs are encouraging for patients with R/R FL and may lead to additional treatment approvals following the approval of mosunetuzumab.

#### Antibody-directed conjugates

The CD79b-directed antibody-directed conjugate (ADC) polatuzumab vedotin combined with rituximab was compared with the anti-CD22 ADC pinatuzumab vedotin plus rituximab in a phase 2 study in patients with R/R NHL [61]. Patients with R/R FL achieved an ORR of 70% (95% CI, 46–88) and a CR rate of 45% (95% CI, 23–68) with polatuzumab vedotin plus rituximab compared with an ORR of 62% (95% CI, 38–82) and a CR rate of 5% (95% CI, 0.1–24) with pinatuzumab vedotin plus rituximab [61]. The most common grade ≥ 3 AEs in patients receiving polatuzumab vedotin plus rituximab were neutropenia (15%) and diarrhea (10%). Clinical studies of additional polatuzumab vedotin combinations are summarized in Table 1. Initial results of a phase 2 study evaluating loncastuximab tesirine, a CD19-directed ADC, combined with rituximab in patients with R/R FL with a median of 1 (range, 1–6) prior line of therapy demonstrated an ORR of 95.2% and a CR rate of 66.7% [62]. Grade 3 AEs included neutropenia (8%), cellulitis (4%), and pleural effusion (4%). Finally, a CD19-directed ADC, tafasitamab, is being evaluated in an ongoing randomized, double-blind, phase 3 study that aims to compare tafasitamab plus R2 with R2 alone in patients with R/R FL or marginal zone lymphoma [63].

#### CAR T-cell therapy

CAR T-cell therapies approved for FL have been discussed previously; however, novel CAR T-cell therapies under investigation in recent and ongoing clinical studies may show promise in the future. In an ongoing phase 2 study of relmacabtagene autoleucel, a CD19 CAR T-cell therapy, in patients with R/R FL, patients with ≥ 2 prior lines of treatment had an ORR of 92.6% (95% CI, 75.1–99.1), with 77.8% (95% CI, 57.7–91.4) achieving CR at the 6-month follow-up [64]. CRS was reported in 42.9% of patients, none of which were grade ≥ 3, and neurotoxicity was reported in 17.9% of patients, of which 1 (3.6%) was grade ≥ 3.

#### BTK inhibitors

Pirtobrutinib, a non-covalent BTK inhibitor, is under investigation in the phase 1/2 BRUIN study (NCT03740529). In 48 patients with R/R FL with a median of 3 (range, 1–12) prior lines of therapy, the ORR was 50.0% (95% CI, 35.2–64.8) and CR rate was 14.6% [65]. The median DOR was 5.5 months (95% CI, 3.7-NE), and median PFS was 5.8 months (95% CI, 3.8–8.1). The most common AEs were diarrhea (29.2%), fatigue (25.0%), and nausea (22.9%). The most common grade ≥ 3 AEs were infection (18.8%) and neutropenia/neutrophil count decreased (14.6%) [65].

### Real-world clinical considerations

Despite several treatment options for FL, both established and novel, treating R/R FL, particularly early or repeated relapses, remains a challenge in the real world. This is partly due to the heterogeneity in FL presentation and patient fitness, leading to diversity in the approach to treatment. Efficacy is an important metric for treatment success; however, in the real world, several patient-specific factors, including the burden of treatment, are considered when deciding on the most appropriate treatment strategy. The rate of disease progression, tumor bulk, and presence of symptoms, as well as patient age, fitness, preference, and cost of treatment, are all factors that affect treatment choice.

Treatment of R/R FL is not required in many cases. When treatment becomes necessary in patients with indolent disease, especially in elderly patients and/or those with comorbidities, a gentler approach is favored and care is taken to minimize harm with aggressive treatments. Choice of treatment should be based on the goals and preferences of the patient after discussion about the pros and cons of each option. With the high median age of patients with relapsed FL after prolonged first remission in the modern era, diagnosis of relapsed disease in patients aged > 75 years is common. As older patients are more likely to experience toxicity with treatment and have altered immune response [66], treatment of this group of patients should be approached with caution and consultation. In addition to dose-reduced and less intensive immunochemotherapy approaches, multiple non-chemoimmunotherapy options can be considered for the majority of patients who have relapsed advanced FL, including R2, EZH2 inhibitors, BTK inhibitors, and BsAbs. Each therapy has a different toxicity profile that needs to be considered, and personalized shared decision-making with patients is essential. R2 is given over a fixed course of 1 year; however, there are dose adjustment considerations for patients with renal failure and cytopenia. Additionally, the tolerable safety profile provided by *EZH2* inhibitors, such as tazemetostat, and the decreased frequency of infusion-related reactions when combining zanubrutinib with obinutuzumab make them attractive in frail patients.

For young and fit patients with a greater tolerance of therapeutic toxicity, or in high-risk patients with rapid progression or early relapse with concerns for occult histologic transformation, a more aggressive approach to treatment is appropriate with consideration about sequencing—including T-cell fitness and the potential use of CAR-T therapies after bendamustine-based approaches. Data from the recent follow-up analysis of the ZUMA-5 study showed that patients with FL with recent bendamustine exposure had worse efficacy outcomes with axicabtagene ciloleucel treatment compared with those with no prior exposure, highlighting the importance of considering prior therapies when choosing treatment options [46]. Novel approaches to treatment may be recommended, including clinical trials, particularly when novel compounds are assessed in earlier lines of therapy. Patient groups such as those with POD24 and poor risk features may benefit from the higher efficacy offered by novel treatment, despite the increased toxicity.

Another potential concern that affects treatment decisions is the uncertain future with COVID-19. Humoral responses to severe acute respiratory syndrome coronavirus 2 (SARS-CoV-2) are slower and less pronounced, and chemoimmunotherapy is associated with lower rates of seroconversion after infection [67]. Response to vaccination is also poor with lymphoma treatments, especially B-cell–depleting therapies with anti-CD20 maintenance [68, 69]. The advent of effective antiviral therapy for COVID-19 has mitigated some of this risk [67]; however, there is still a risk of symptomatic COVID-19 infection with novel B-cell lymphoma treatments, particularly those that result in prolonged B-cell depletion.

In the real world, adequate access to novel treatments may be a concern for patients with FL. Advances in the development of CAR T-cell therapy provide new treatment options for patients with R/R FL; however, several barriers limit access to this therapy. Patients may not be eligible for CAR T-cell therapy, and the cost of CAR T-cell therapy makes it an unlikely option outside of clinical trials for those without adequate health insurance coverage. Considering logistical barriers, eligibility, and administration methods, more widely available, financially accessible, and well-tolerated treatment options may be chosen instead. BsAbs, particularly mosunetuzumab, are immediately available off-the-shelf agents with growing experience in administration and mitigation of infusion reactions.

Similarly, ZO, R2, and tazemetostat are more easily administered in the ambulatory care setting, which is of value for patients who would prefer outpatient administration over frequent infusion visits or hospitalizations and patients with symptomatic relapse in need of immediate treatment within the waiting period for CAR T-cell therapy.

### Unmet needs and future direction

With so many treatment options available for FL, determining the best approach to therapy of R/R FL is challenging. This challenge is compounded by the diversity of clinical presentations of FL, including a significant minority with early progression or histologic transformation. Radiological evidence, such as a very high standardized uptake value with ^18^fluoro-2-deoxyglucose–positron emission tomography, may be predictive of histological transformation, which should be confirmed by a biopsy directed at the site of high standardized uptake value [70]. Additionally, high lactate dehydrogenase levels may be associated with a higher risk of transformation [71]. Identifying or ruling out histologic transformation early is important for adjusting treatment choices to improve patient prognosis.

POD24 remains the most robust marker of poor outcome in patients with FL, but even this population has significant heterogeneity in outcome, and predicting future POD24 is an ongoing challenge. Patients with POD24 require a unique approach to treatment, including the use of novel therapies. A study of measurable residual disease (MRD) as a prognostic marker in FL has demonstrated a significant association between POD24 and MRD status during induction treatment; however, the use of MRD status was better for excluding POD24 than for predicting it [72]. Although this association may be valuable in predicting POD24 early in a small proportion of patients, further development of consistent markers for prospective identification of early progression and treatment failure is still required to improve outcomes in high-risk patients with FL.

A further challenge in practice is optimal sequencing of therapies for patients with R/R FL, especially as incorporation of new agents into earlier lines of therapy will continue to influence the choice and sequencing of subsequent therapies. With several novel therapies in development, it is unlikely that established treatments such as rituximab or obinutuzumab plus CHOP, BR, or bendamustine plus obinutuzumab will be studied as second-line therapies, but they are likely effective options in practice. Additionally, to address the unmet needs of patients with multiple relapses, several ongoing trials are evaluating novel treatment options. As of May 9, 2024, a search of clinicaltrials.gov found 184 ongoing clinical trials in the “recruiting” or “active, not recruiting” stages for R/R FL. The high volume of ongoing trials demonstrates the need for novel treatment options in this therapeutic area, while also suggesting the potential for new and effective treatments in the future.

## Conclusions

Recent developments in treating FL have resulted in favorable outcomes in most patients. Patients often survive for decades, and many are functionally cured. However, lymphoma remains the most common cause of death in patients with FL. Novel therapeutic approaches in development offer clinicians a wide array of options in an R/R setting. Challenges arise in the form of select groups of patients who have early progression or are unable to tolerate the toxicity of aggressive or more immune-suppressive treatments. Nuanced decisions accounting for both risks and benefits of each treatment option need to be made to address the needs of individual patients.

## Data Availability

No datasets were generated or analyzed during the current study.

## Abbreviations

ADC: antibody-directed conjugate
AE: adverse event
BR: bendamustine plus rituximab
BsAb: bispecific antibody
BTK: Bruton tyrosine kinase
CAR: chimeric antigen receptor
CHOP: cyclophosphamide, doxorubicin, vincristine, and prednisone
CR: complete response
CRS: cytokine release syndrome
DOR: duration of response
FL: follicular lymphoma
FLIPI: Follicular Lymphoma International Prognostic Index
HMT: histone methyltransferase
ICANS: immune effector cell–associated neurotoxicity syndrome
MRD: measurable residual disease
NE: not evaluable
NHL: non-Hodgkin lymphoma
ORR: objective response rate
OS: overall survival
PFS: progression-free survival
PI3K: phosphoinositide 3-kinase
POD24: progression of disease within 24 months of first-line treatment
R/R: relapsed/refractory
R2: rituximab plus lenalidomide
SCT: stem cell transplant
ZO: zanubrutinib plus obinutuzumab
